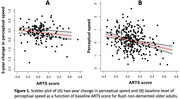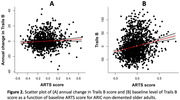# The ARTS Marker of Arteriolosclerosis: Instrumental and Clinical Validation

**DOI:** 10.1002/alz.086591

**Published:** 2025-01-09

**Authors:** Konstantions Arfanakis, Arnold M Evia, Sue E. Leurgans, Myriam Fornage, Hanzhang Lu, Caprihan Arvind, Charles Decarli, Brian T Gold, Pauline Maillard, Claudia L Satizabal, Lara Stables, Danny JJ Wang, Karl G Helmer, Bruce Fischl, Herpreet Singh, Kristin Schwab, Steven M. Greenberg, Julie A. Schneider, MarkVCID Consortium

**Affiliations:** ^1^ Rush University Medical Center, Chicago, IL USA; ^2^ Illinois Institute of Technology, Chicago, IL USA; ^3^ The University of Texas Health Science Center at Houston, Houston, TX USA; ^4^ Johns Hopkins University School of Medicine, Baltimore, MD USA; ^5^ The Mind Research Network, Albuquerque, NM USA; ^6^ University of California, Davis, CA USA; ^7^ University of Kentucky, Lexington, KY USA; ^8^ Department of Neurology and Center for Neuroscience, University of California, Davis, CA USA; ^9^ The University of Texas Health Science Center at San Antonio, San Antonio, TX USA; ^10^ University of California, San Francisco, CA USA; ^11^ University of Southern California, Los Angeles, CA USA; ^12^ Massachusetts General Hospital, Boston, MA USA

## Abstract

**Background:**

The ARTS biomarker is a fully automated software container that predicts the presence of arteriolosclerosis based on in‐vivo MRI data and demographic features. The present study describes findings from the instrumental and clinical validation of ARTS conducted by the MarkVCID consortium.

**Method:**

Instrumental validation of ARTS involved assessment of inter‐rater reliability, test‐retest repeatability, and inter‐scanner reproducibility. Inter‐rater reliability was assessed by means of the intraclass correlation (ICC) between ARTS scores generated from all MarkVCID sites using data on 20 older adults. Test‐retest repeatability was assessed by means of the ICC between test and retest ARTS scores from 41 older adults imaged twice. Inter‐scanner reproducibility was assessed by means of the ICC between ARTS scores on 20 older adults imaged on 4 different scanners.

Clinical validation of ARTS included two groups of non‐demented older adults: N=156 participating in community cohort studies at Rush Alzheimer’s Disease Center; and N=906 participating in the Atherosclerosis Risk in Communities (ARIC) study. The primary prespecified hypothesis was that change in Trails B score (or perceptual speed score for Rush data) two years after baseline MRI is associated with baseline ARTS score, controlling for years of education. The secondary prespecified hypothesis was that baseline Trails B (or perceptual speed) is associated with baseline ARTS score, controlling for years of education.

**Results:**

ARTS exhibited excellent inter‐rater reliability (ICC=0.9999, p<10^‐200^, CI:[0.99987, 0.99997]), test‐retest repeatability (ICC=0.996, p<10^‐42^, CI:[0.993, 0.998]), and inter‐scanner reproducibility (ICC=0.955, p=0.00018, CI:[0.622,0.989]). In Rush data, a higher baseline ARTS score was associated with a faster two‐year decline in perceptual speed (beta=‐0.32, p=0.04, CI:[‐0.63, ‐0.01]) (Figure 1A) and a lower baseline level of perceptual speed (beta=‐1.04, p<10^‐4^, CI:[‐1.48, ‐0.60]) (Figure 1B). In ARIC data, a higher baseline ARTS score was associated with a faster annual increase in Trails B (beta=3.7, SE=1.7, p=0.03) (Figure 2A), and a higher baseline Trails B (beta=45.5, SE=5.8, p<10^‐4^) (Figure 2B).

**Conclusion:**

ARTS exhibited outstanding technical performance and baseline ARTS score was associated with subsequent decline in cognitive abilities known to be affected by small vessel disease. The above evidence supports ARTS as a marker of the risk of vascular contributions to cognitive impairment and dementia (VCID).